# Research Hotspots and Trend Exploration on the Clinical Translational Outcome of Simulation-Based Medical Education: A 10-Year Scientific Bibliometric Analysis From 2011 to 2021

**DOI:** 10.3389/fmed.2021.801277

**Published:** 2022-02-07

**Authors:** Shun Yao, Yabin Tang, Chenyue Yi, Yao Xiao

**Affiliations:** ^1^Clinical Skills Training Center, Zhujiang Hospital, Southern Medical University, Guangzhou, China; ^2^Department of Neurosurgery, Huashan Hospital, Shanghai Medical College, Fudan University, Shanghai, China

**Keywords:** simulation-based medical education (SBME), clinical skill, translational outcomes, bibliometric, scientific visualization analysis

## Abstract

**Background:**

In recent decades, an increasing number of studies have focused on the clinical translational effect of simulation-based medical education (SBME). However, few scientific bibliometric studies have analyzed the research hotspots and publication trends. This study aimed to investigate research hotspots and future direction in the clinical translational outcome of SBME via bibliometrics.

**Method:**

Relevant publications on the clinical translational outcomes of SBME from 2011 to 2021 were identified and retrieved from the Web of Science Core Collection (WOSCC). Software including VOSviewer (1.6.17) and CiteSpace (5.8R3) and a platform (bibliometric.com) were employed to conduct bibliographic and visualized analysis on the literature.

**Results:**

A total of 1,178 publications were enrolled. An increasing number of publications were observed in the past decades from 48 in 2011 to 175 in 2021. The United States accounted for the largest number of publications (488, 41.4%) and citations (10,432); the University of Toronto and Northwestern University were the leading institutions. *Academic Medicine* was the most productive journal concerning this field. McGaghie W C and Konge L were the most influential authors in this area. The hot topic of the translational outcome of SBME was divided into 3 stages, laboratory phase, individual skill improvement, and patient outcome involving both technical skills and non-technical skills. Translational research of comprehensive impact and collateral outcomes could be obtained in the future.

**Conclusion:**

From the overall trend of 10 years of research, we can see that the research is roughly divided into three phases, from laboratory stage, individual skill improvement to the patient outcomes, and comprehensive impacts such as skill retention and collateral effect as cost-effectiveness is a major trend of future research. More objective evaluation measurement should be designed to assess the diverse impact and further meta-analysis and randomized controlled trials are needed to provide more clinical evidence of SBME as translational science.

## Introduction

Simulation-based medical education (SBME), first proposed in the 1970s (1), has made rapid progress in the past 50 years. Unlike primary skill acquisition, SBME provides a visual specific scene for trainees to mimic clinical scenes, aiming to improve medical practice and patient outcomes directly (2, 3). Considering that “deliberate practice” is vital in medical education and patients' demand for skillful doctors and comfortable clinical services, the advantages of SBME are as follows: (i) it provides immediate feedback; (ii) it adapts to the needs of teaching, allowing deliberate and repeated training; (iii) it offers medical students and practitioners a safe and standard platform to assess and improve skills (2). SBME has been acknowledged to have positive effects on medical skills acquisition and proficiency compared with traditional clinical education (4). As simulators and training methods improve, considerable evidence shows the utility and effectiveness of SBME, including in various medical specialties, not only in technical skills but also in non-technical skills. However, simulated training based on deliberate practice was still in its initial stage (5–7). It was not until 2010 that McGaghie first proposed the research on SBME as a concept of translational science research (8), suggesting a bright new stage of SBME research: translation to the improvement of clinical skills and patient outcomes. So far, few studies have analyzed the research hotspots and future publication trends in this field.

Bibliometrics can provide a general picture and shows the developing trends in one specific research field and identifies the most influential elements, including the institutions, authors, journals, emerging keywords, etc; the method can also show the research direction and emerging topics in a scientific domain (9, 10). Recently, an increasing number of bibliometric studies of knowledge synthesis have been published due to the explosive and rapid production of outputs, the rapid updating of knowledge, and the development of computer technology. Many methods of knowledge synthesis and software in a digital machine-readable format were developed (11), which were even used in the medical area (12, 13). Based on a knowledge synthesis methodology, such as the triangulation of distant reading from recent research (14, 15), we used visualization and bibliometric analysis with the bibliometric software such as VOSviewer, and CiteSpace to explore the hotspots and current status of translational research of SBME from 2011 to 2021, as well as to guide the direction of scholars in this field.

## Methods

### Data Collection

**Figure 1:**
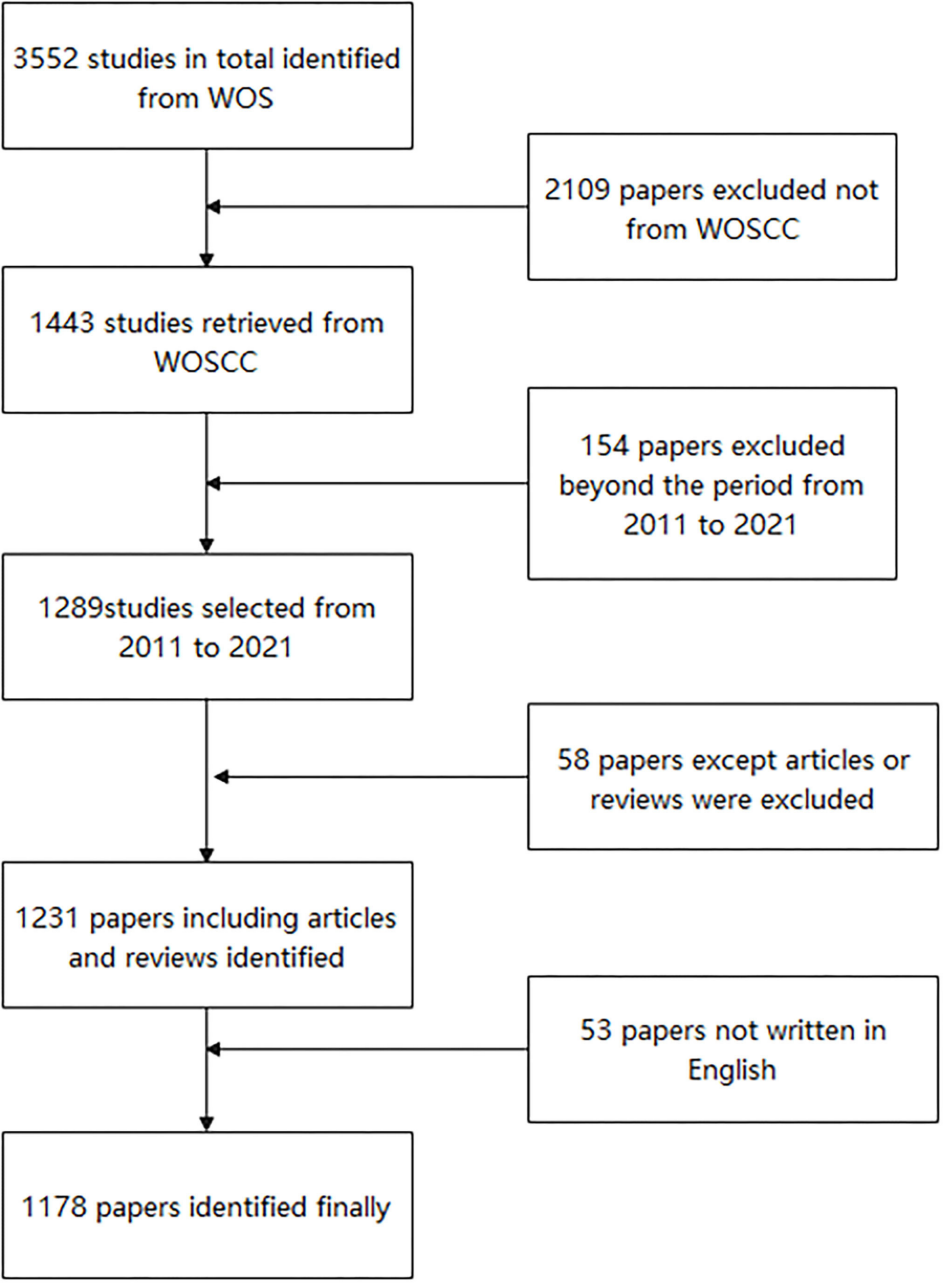
Flow diagram of the inclusion process. The detailed process of data collection and selection.

We retrieved the data from the Web of Science Core Collection (WoSCC) to acquire publications concerned with the translational outcome of SBME since WOSCC was considered the optimal database which is most used in the existing bibliometric analysis. The search strategies were as follows: TS = (simulation-based medical education OR simulation-based training OR medical simulation training) AND TS = (clinical skills OR technical skills OR patient-related outcomes) AND TS = (transfer OR translational outcome OR improve). All searches were performed on a single day, December 6, 2021, to avoid bias produced by daily database renewal. A total of 1,178 records published from January 1, 2011–December 6, 2021 were obtained for further study including only Articles and Reviews. all written in English. Detailed selective procedures of the enrollment and screening were illustrated as a flowchart in Figure 1.

### Software and Data Analysis

All the publications included were exported in TEXT format and then imported into CiteSpace (5.8.R3), VOSviewer (1.6.17), and a bibliometric platform *(bibliometric.com)* for data visualization. Basic information, such as the yearly output and citation frequency was obtained from the WoSCC. We next analyzed the trends of publication of each country and drew a cooperative network of the countries on the website platform. In this study, the frequency was used to descript the number of institutions, journals, and keywords with CiteSpace. Different nodes in the visualization represent different institutions or keywords. A line between nodes refers to a cooperative network. Regarding the selection of time slices, a slice of 1 year was used because of the higher modularity value and the silhouette value of the clustering effect. Regarding the connection strength, cosine was used. Regarding the threshold, we selected the top 50 nodes at each time point. Moreover, the pruning used pathfinder and the merged network (16). By adjusting corresponding parameters, cluster analysis and timeline view were performed for keywords and keyword burstiness was performed to explore the emerging topics and future direction (17). Moreover, we used the VOSviewer to illustrate the co-authorship and its co-occurrence analysis. The number of publications and collaborations among authors could be seen from the overlay visualization map of the author's co-authorship analysis. The same color represented a closely related cluster of authors; the size of the circle represented the number of publications, and the thickness of the line represented the closeness of cooperation between the authors.

## Results

### Global Publications and Citations

**Figure 2:**
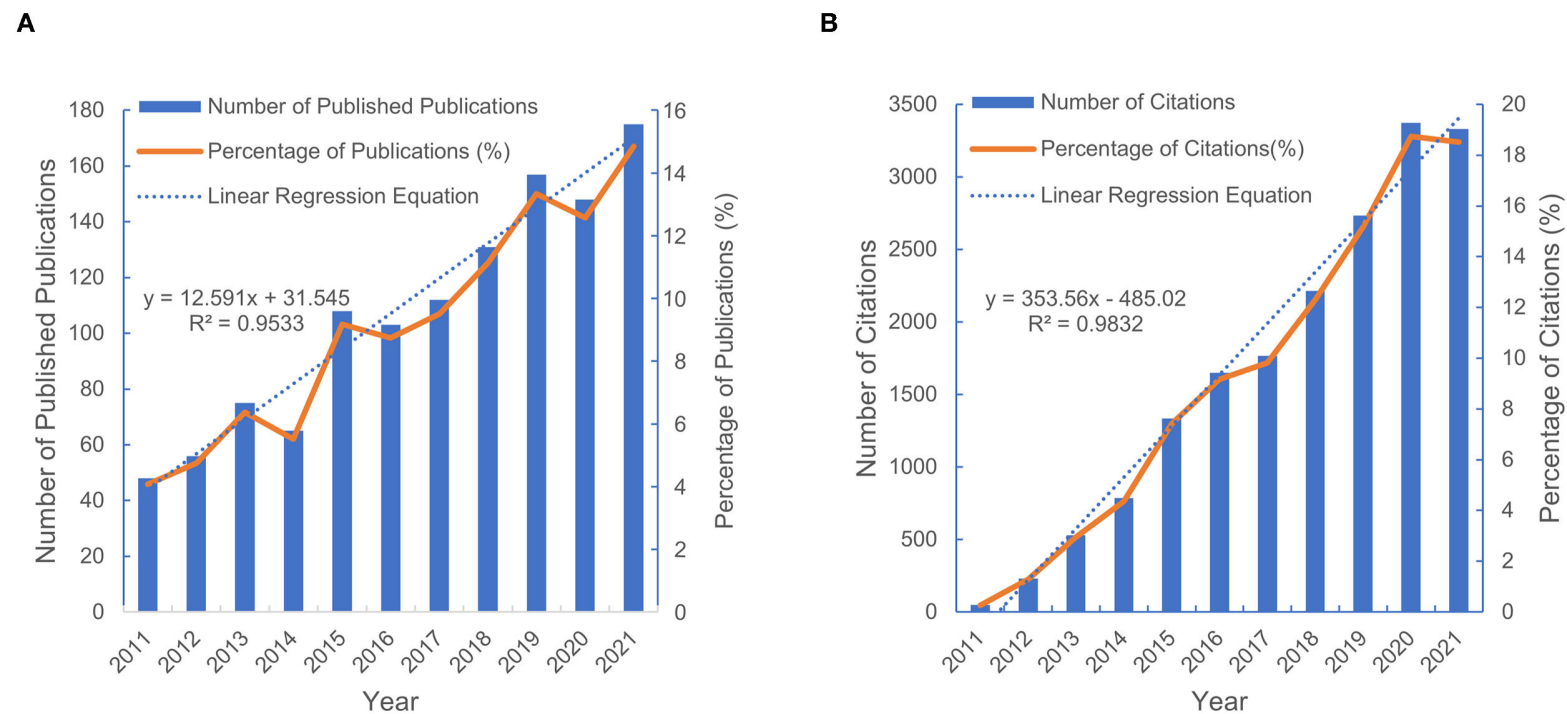
Global number of publications and citations from 2011 to 2021. **(A)** Annual number of the published publications and its percentage in the total publications. **(B)**Number and percentage of the annual publication citations.

An increasing trend in the number of publications was observed in the past decade, from 48 in 2011 to 175 in 2021. Figure 2A exhibited an overall upward trend in the research of translational outcome of SBME, especially from 2014 to 2015, during which time McGaghie W C put forward the translational outcome paradigm shift of SBME. Notably, the linear fitting of the studies shows a significant positive correlation (*R*^2^ = 0.9533) between the year and the number of publications. We conservatively estimated that the number of papers published in 2022 will be more than 185. Additionally, the publication citations also showed a continuous growth trend (Figure 2B), consistent with the linear growth curve model (*R*^2^ = 0.9832).

### Distribution Characteristics of Countries and Institutions

**Figure 3:**
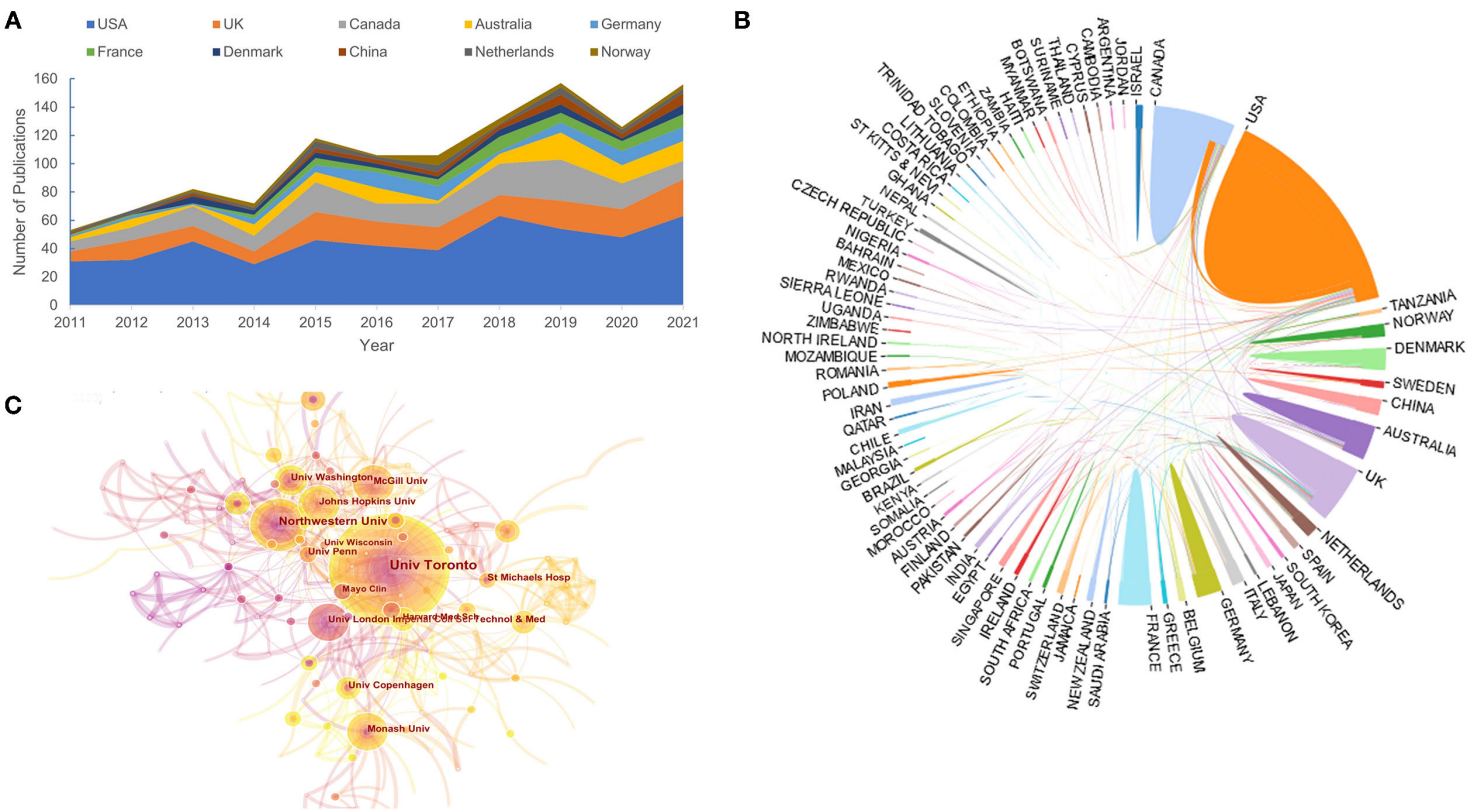
Distribution characteristic of countries and institutions. **(A)** Trends of annual publications of 10 most productive countries. **(B)** International collaboration between countries. The countries are labeled using different colors and the links represents international collaborations. **(C)** Institution collaboration network formed by CiteSpace.

Analysis of the county or institution distribution of publications can provide great information for researchers about the countries or institutions at the leading frontiers of research. In our study, we identified the 10 most productive countries and institutions involved in this research (Table 1). The USA was far ahead of other countries, with 488 publications accounting for 41.4% of the total, followed by Canada (170, 14.4%), the UK (160, 13.6%), and Australia (92, 7.8%). Furthermore, the USA participated most frequently in international cooperation, followed by Canada, the UK, Australia, and Denmark (Figures 3A,B).

**Table 1:** The top 10 countries with the highest number of publications and citations.

**Rank**	**Country**	**Article counts**	**Total number of citations**	**Average number of citations**
1	USA	488	10,432	21.38
2	Canada	170	3,253	19.14
3	UK	160	3,072	19.2
4	Australia	92	1,461	15.88
5	Germany	62	597	9.63
6	France	49	305	6.22
7	Denmark	35	591	16.89
8	Netherlands	30	497	16.57
9	Norway	28	574	20.50
10	Italy	28	173	6.18

We also identified the top 10 institutions shown in Table 2. Among them, 40% of institutions were from the United States, 30% were from Canada and only three belonged to Australia, Denmark, and the UK individually, in line with the distribution characteristics of countries. Based on the collaboration network formed by CiteSpace (Figure 3C), the University of Toronto and Northwestern University have the highest total link strength, indicating that these two institutions participated in most collaborations with other institutions worldwide. With extensive academic communication among scholars, it was necessary to develop closer research collaboration between various institutions for the lower level.

**Table 2:** The top 10 institutions with the highest number of publications.

**Rank**	**Institution**	**Article counts**	**Total number of citations**	**Average number of citations**	**Country**
1	Univ Toronto	76	1,445	19.01	Canada
2	Northwestern Univ	38	2,253	59.29	USA
3	McGill Univ	26	441	16.96	Canada
4	Univ Washington	25	715	28.60	USA
5	Monash Univ	25	425	17.00	Australia
6	Univ Penn	22	553	25.14	USA
7	Johns Hopkins Univ	21	355	16.90	USA
8	St Michaels Hosp	21	516	24.57	Canada
9	Univ Copenhagen	21	281	13.38	Denmark
10	Univ London Imperial Coll Sci Technol & Med	21	830	39.52	UK

### Journals Publishing Research on the Translational Outcome of SBME

There were 475 journals in total that published research involved in this field with the top 10 listed in Table 3. More than one-fifth (23%) of the publications in this field were published in the listed top 10 journals. *BMC Medical Education* and *Journal of Surgical Education* were the top two journals that published the same number of papers (*n* = 53) and the total number of citations of them were 388 and 882, respectively. It was obvious that these 10 journals laid a solid foundation for subsequent research on SBME. The average number of citations and the impact factor were also important indicators for evaluating the influence of journals. Medical Education (66.75) and Academic Medicine (55.54) had the highest average number of citations, in line with the order of impact factors according to the 2021 JCR standards.

**Table 3:** The top 10 journals contributing to publish articles in spinal stenosis research.

**Rank**	**Journal**	**Article counts**	**Percentage %**	**Total number of citations**	**Average number of citations**	**IF (JCR 2021)**	**Quartile in category (JCR)**	**H-index**
1	BMC medical education	53	4.50	388	7.32	2.463	Q1	68
2	Journal of surgical education	53	4.50	882	16.64	2.891	Q1	54
3	Simulation in healthcare-journal of the society for simulation in healthcare	48	4.07	977	20.35	1.929	Q3	–
4	Surgical endoscopy and other interventional techniques	23	1.95	410	17.82	4.584	Q1	152
5	BMJ simulation & technology enhanced learning	21	1.78	21	1.00	–	Q4	9
6	Cureus	21	1.78	39	1.86	–	Q3	–
7	American journal of surgery	14	1.19	340	24.28	2.565	Q2	153
8	Clinical simulation in nursing	14	1.19	124	8.86	2.391	Q1	41
9	Academic medicine	12	1.02	1191	55.54	6.893	Q1	152
10	Medical education	12	1.02	801	66.75	6.251	Q1	138

### Authors and Co-authorship Analysis

**Figure 4:**
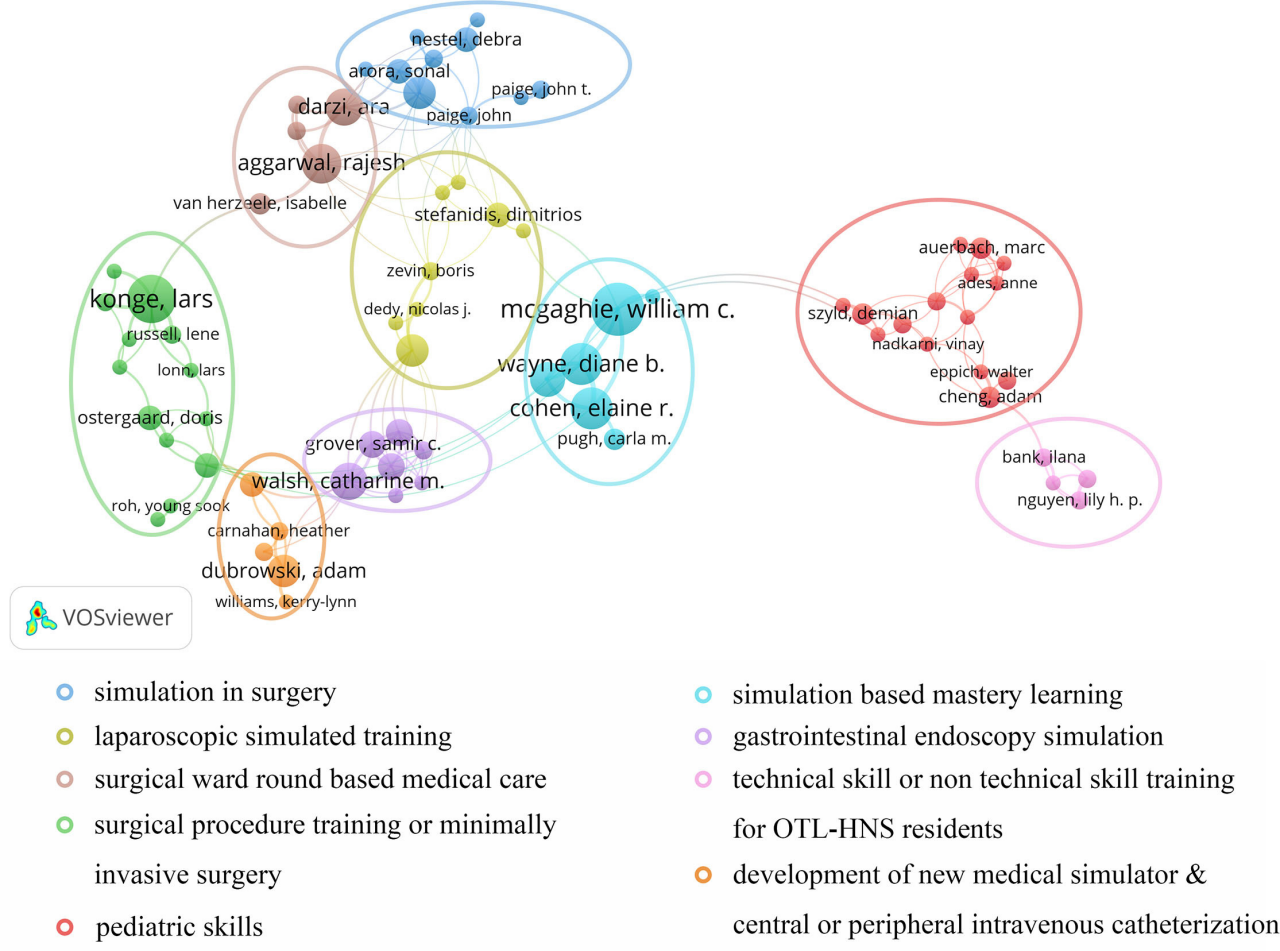
VOSviewer network visualization map of co-cited authors. Authors collaborated to study different fields of SBME were circled by different colors.

A total of 5,975 authors were involved in current work according to VOSviewer analysis but only 133 authors met the minimum publication thresholds when set to 3. The top 10 most productive authors were shown in Table 4. Nearly half were from the USA, including three belonging to Northwestern University. Meanwhile, by analyzing the articles in each cooperation cluster, we manually and subjectively identified nine clusters and labeled them in areas of research for each cluster with different colors as Figure 4.

**Table 4:** The top 10 most productive authors.

**Rank**	**Author**	**Article counts**	**Instituition**	**Total number of citations**	**Country**
1	McGaghie WC	18	Northwestern Univ	1,640	Canada
2	Konge L	17	Univ Copenhagen	157	USA
3	Aggarwal R	14	Univ London Imperial Coll Sci Technol & Med	348	Canada
4	Cohen ER	14	Northwestern Univ	1,155	USA
5	Wayne DB	14	Northwestern Univ	1,398	Australia
6	Darzi A	11	Univ London Imperial Coll Sci Technol & Med	233	USA
7	Walsh CM	11	Univ Toronto	185	USA
8	Barsuk JH	10	Northwestern Univ	1,274	Canada
9	Grantcharov TP	10	St Michaels Hosp	368	Denmark
10	Sevdalis N	10	Univ London Imperial Coll Sci Technol & Med	535	UK

### Keyword Cluster and Burstiness

**Figure 5:**
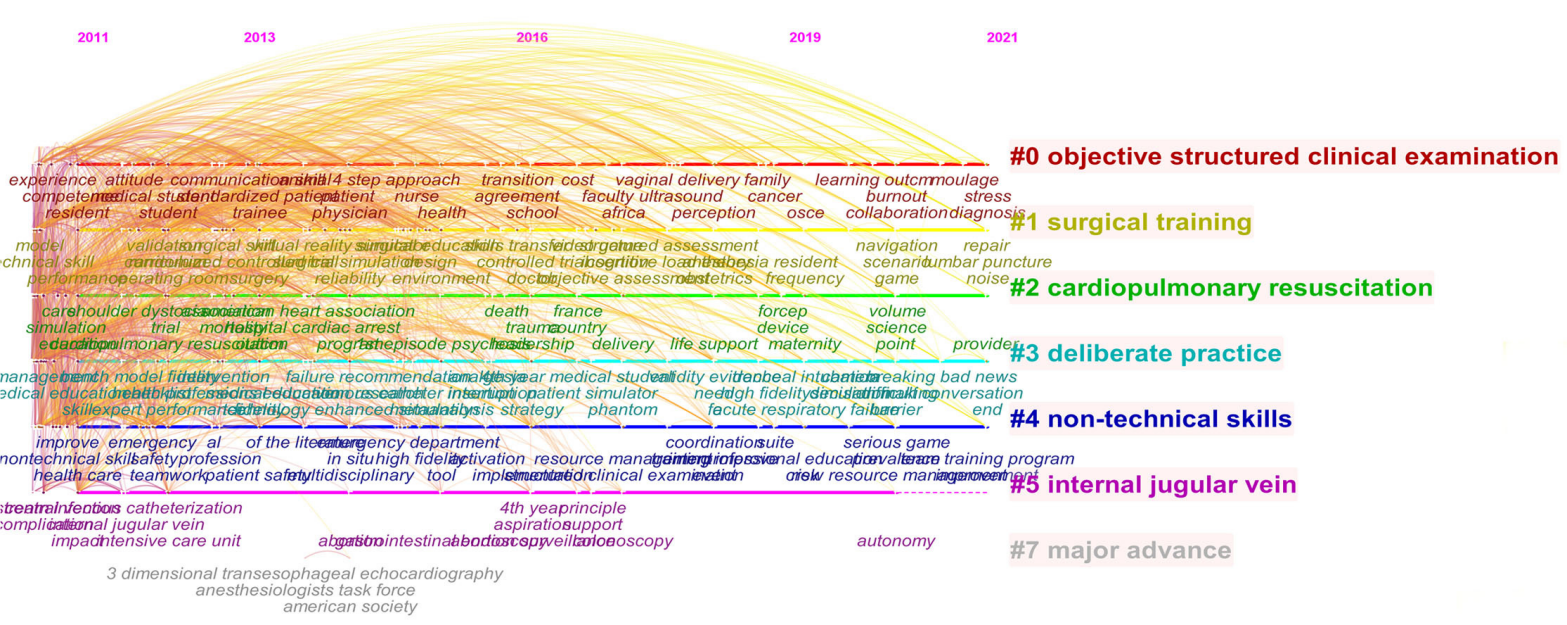
Timeline visualization from 2011 to 2021. Nodes are labeled with corresponding topics.

**Figure 6:**
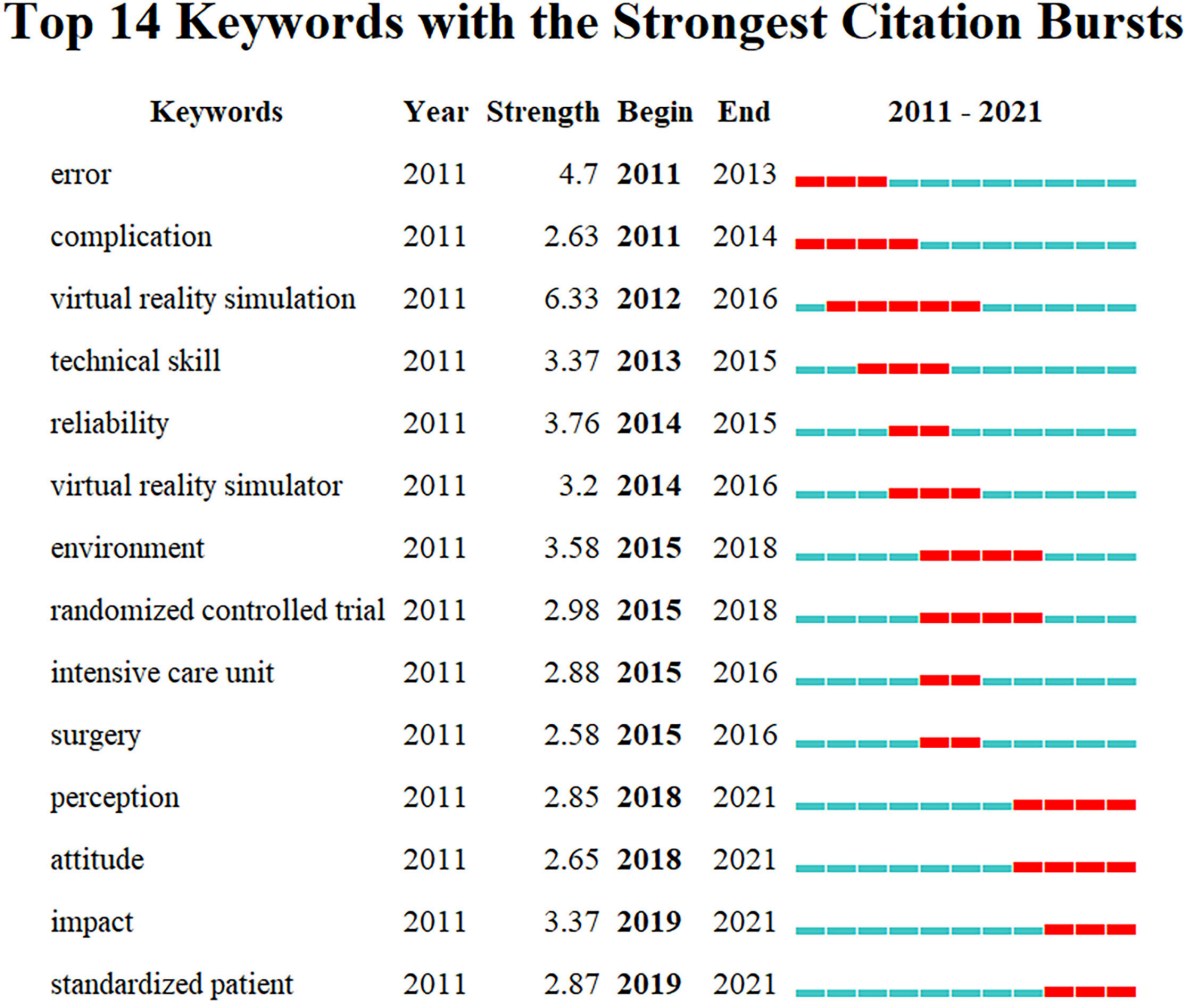
Top 14 keywords with strong citation burstness. The red bars meant some references cited frequently; the blue bars were the time interval when references cited infrequently.

Cluster for research hotspots was identified with keyword co-occurrence by CiteSpace (5.8.R3). Seven clusters in total were formed including objective structured clinical examination, surgical training, cardiopulmonary resuscitation, deliberate practice, non-technical skills, internal jugular vein, and major advances. Timeline view was used to depict a timeline for keywords after clustering and the length of the horizontal line corresponding to each cluster represents the span of the cluster (Figure 5). CiteSpace was further used to detect bursts of keywords with high frequency (Figure 6).

### Analysis of the Top Ten Most Highly Cited References

Published papers that are cited frequently possess tremendous academic impact. Table 5 shows the top 10 articles that had been highly cited in the translational outcome of SBME studies. Most of the highly cited articles in the table were research articles published in authoritative journals with high impact factors such as JAMA (IF = 56.272, Q1), Journal of the American College of Cardiology (IF = 24.094, Q1), BMJ Quality & Safety (IF = 7.035, Q1).

**Table 5:** The top 10 high-cited references.

**Rank**	**Title**	**Author**	**Citation**	**Journal**	**Year**
1	Does simulation-based medical education with deliberate practice yield better results than traditional clinical education? a meta-analytic comparative review of the evidence	McGaghie WC	770	Academic medicine	2011
2	A critical review of simulation-based mastery learning with translational outcomes	McGaghie WC	257	Medical education	2014
3	Effect of communication skills training for residents and nurse practitioners on quality of communication with patients with serious illness a randomized trial	Curtis JR	242	JAMA	2013
4	Cognitive interventions to reduce diagnostic error: a narrative review	Graber ML	220	BMJ quality & safety	2012
5	Simulation-based mock codes significantly correlate with improved pediatric patient cardiopulmonary arrest survival rates	Andreatta P	218	Pediatric critical care medicine	2011
6	Systematic review of skills transfer after surgical simulation-based training	Dawe SR	206	British journal of surgery	2014
7	Teamwork and leadership in cardiopulmonary resuscitation	Hunziker S	160	Journal of the American college of cardiology	2011
8	*In situ*, multidisciplinary, simulation-based teamwork training improves early trauma care	Steinemann S	154	Journal of surgical education	2011
9	Evaluating the impact of simulation on translational patient outcomes	McGaghie WC	151	Simulation in healthcare-journal of the society for simulation in healthcare	2011
10	Non-technical skills training to enhance patient safety: a systematic review	Gordon M	136	Medical education	2012

## Discussion

### Current Status in SBME Research as Translational Science

#### General Information

In this study, 1,178 publications originating from the WoSCC were analyzed, aiming to explore the hotspots and development trends of the research on the translational outcomes of SBME from 2011 to 2021. For the first time, bibliometric analysis and visualization mapping were utilized in this research field. The number and trends of publications and citations showed increasing research focus. With the development of advanced simulators and progress in medical education, this field will probably remain a hotspot in the next few decades as it provides a beneficial solution to the growing demand for skillful doctors and public health services. As was shown in country distribution, the USA was the most influential country and had the most frequent cooperation with other countries. Among the top 10 most productive institutions, four were located in the USA. Notably, Northwestern University was the second most productive institution. Before 2010, a team from Northwest University under McGaghie published three critical reviews to summarize the historical trends of simulation-based medical education (2, 18, 19). Firstly, the emergence of medical simulators has laid the foundation of medical simulation education. In the 1990s, high-technology simulations appeared in four medical areas: laparoscopic, cardiovascular disease simulator, multimedia computer systems, and anesthesia simulators. In 2005, the team published the first best evidence medical education (BEME) systematic review of the research evidence on the features and use of high-fidelity medical simulations that lead to effective learning. They then published another critical review of SBME in 2003–2009, identified twelve features and best practice methods. It was not until 2010 that McGaghie first claimed SBME research as translational science, indicating the beginning of the research explosion toward the clinical translational outcomes of SBME (8). Therefore, the concept “translational science” in this area was first expressly proposed by Northwestern University. So it was not difficult to understand why the USA lead in this area. In addition, the University of Toronto, another core center in cooperative mapping, had the most published outputs, revealing that Canada is developing rapidly in this field. Other countries listed in Table 1 are all developed countries while no Asian countries were found. At the same time, geographical imbalance in research was also reflected, suggesting that the strength of medical education in developing countries is still lacking.

Remarkably, journals in the field of SBME translational research such as the *BMC Medical Education, Journal of Surgical Education*, and *Simulation in Healthcare-Journal of the Society for Simulation in Healthcare* were the primary journals involved in the publication. Therefore, it is reasonably concluded that future developments in this field are more likely to be published in these journals.

#### Co-authorship Collaborative Cluster

In addition to listing the top 10 productive authors, we also identified the co-authorship cluster visualization to analyze the collaborative orientation. The red cluster is divided into two sub-groups centered on Nishisaki et al. (20) and Cheng et al. (21) with orientations concerned pediatric skills. The former proved that simulation-based training improved the performance of team behavior during tracheal intubation procedures as well as pediatric advanced life support in Intensive Care Unit (ICU) (22), while the latter found great effectiveness of SBME translated into cardiopulmonary resuscitation (CPR) in pediatric emergency medicine. The green cluster represented the orientations of various catheter intubation and various surgical training including flexible fiberoptic intubation and mastoidectomy (23–25). Notably, the central author of this cluster, in Nilsson et al. (26), also paid great attention to laparoscopy, cystoscopy, and procedures guided by ultrasound, which made a great contribution to the translation effect of minimally invasive surgery (27–29). Blue and yellow clusters centered on Zevin et al. (30) and Sevdalis et al. (31) concentrated on surgically similar translational outcomes especially laparoscopic surgery. Moreover, the purple cluster mainly focused on gastrointestinal endoscopy, particularly technical or non-technical skill translation of colonoscopy (32–34). Two other clusters worth mentioning in particular were the brown cluster and the pink cluster, mainly discussing upgrading skills based on surgical ward care (35) and non-technical skill translation effects in the department of otolaryngology and head-neck surgery (OTL-HNS). Although ward rounds are not currently subject to formal training, they are an extremely crucial skill. Pucher et al. recommended the implementation of a comprehensive curriculum for surgical ward rounds inducing significant improvement in the quality of patient assessment, management, and non-technical skills which may lead to earlier identification and amelioration of complications and improve patient outcomes (36). Furthermore, Young M focused on the clinical transformation of sinus surgery skills training as well as skill development in communication and leadership for OTL-HNS residents (37, 38).

### Research Focused on the Translational Outcome of SBME

#### Influential Research

Published papers that are cited frequently produce enormous academic influence. Among the top 10 highly-cited references (Table 5), three publications consolidated the important position of translational research in SBME, written by McGaghie et al. who was the most productive author in this area. The most cited paper published in *Academic Medicine* in 2011 has been cited 770 times, which emphasized the importance of deliberate practice (DP) before achieving specific clinical skill acquisition goals suggesting an initial but essential pathway to realize the translational outcome in nearly all clinical skills (4). Another two references by McGaghie et al. are also the most comprehensive review about SBME as translational science until now. The one published on *Medical Education* in 2014 emphasized the significance of translational feature of simulation-based mastery learning (SBML) that can produce downstream results, indicating innovative ideas for future research (39). The other cited 151 times proposed that rigorous SBME translational science research can contribute to better patient care and improved patient safety (40). We can see from the list that the translational research of simulated skill training mainly focused on two aspects of specialties, technical skills, and non-technical skills. Referring to technical skills, Andreatta P designed a simulation-based mock code that proved effective in improving the response time of residents when providing CPR and correlates with the enhancement of the CPR clinical skill (41). Referring to non-technical skills, *Journal of the American College of Cardiology, Journal of Surgical Education, Medical Education, BMJ Quality & Safety* published four papers related to teamwork (42, 43), leadership training (43), and cognitive intervention (44) that enhances the clinical performance (45). However, the paper published on *JAMA* in 2013 presented an opposite view that among physicians and nurses, simulation-based communication training does not improve the quality of communication on hospice care or quality compared with traditional education (46). The non-technical skills of the human factors gradually come into prominence but their translational effects after SBME are still unclear, more improvement in teaching practice and the creation of effective teaching methods are needed.

#### Research Focuses Changed Greatly Over Time

According to the timeline view (Figure 5) and burst test of keywords (Figure 6), combined with the number of chronological documents, the research on the translational outcome of SBME can be roughly divided into three stages, in line with at least three seamless phases of translational science progress from bench to bed.

The first stage is from 2011 to 2014, corresponding to the T1 stage of translational science: educational laboratory. At this stage, translational outcomes are educational effects measured at increasingly distal levels beginning in a classroom or medical simulation laboratory and initially moving downstream. Laboratory training can effectively improve residents' ability to perform anastomoses, which may result in increased efficiency of teaching in the operating room. For example, Burton found simulation-based training is an effective method to improve safety knowledge, attitudes, and teamwork surrounding extracorporeal membrane oxygenation (ECMO) emergencies in the simulation laboratory environment. Sarwani et al. (47) and Sonnadara et al. (48) also explored the laboratory-based simulation courses in Acute Radiologic Emergencies and surgery. Meanwhile, researchers also focused on finding some objective structured clinical measurement or examination to reflect the improving practice such as operating error, complication, operating time, and pathway length (44, 49). Future progress will require methodological refinements in outcome evaluation and rigorous evaluation on interventions already suggested, many of which such as practice error and complications are well-conceptualized and widely endorsed. With the completion of simulated medical education facilities in recent years, the simulated medical training laboratory transformation in laparoscopic surgical suture has achieved great results (50). Clinical diagnostic medicine is gradually establishing laboratory conditions to simulated skills training, which is the research hotspot of laboratory simulation-based medical education at present (51).

The second stage covers the period from 2015 to 2018 corresponding to the T2 stage of translational science: improved patient care practices, which is the downstream of T1. During this time, individual clinical skill improvement after simulated training has been gradually proved in both technical skills or non-technical skills and an increasing number of randomized controlled trials are carried out to provide clinical evidence. For example, in the field of surgical skills, one of the most progressive areas of translational research of SBME, Jonathan found that after simulation-based training, residents perform vascular and bowel anastomoses more adeptly, quickly, and with a higher quality (52). Another two technical skills improvements worth mentioning are central venous catheterization and CPR in ICU and emergency medicine (21, 53). At the same time, SBME of non-technical skills such as communication practice, teamwork, and leadership practice are also proven to be potentially translated to clinical skill performance (54, 55).

In the third phase of 2018–2021, translational research on SBME seems to focus on the advanced patient outcomes and patient safety which correspond to the T3 phase of translational science: patient outcome, not only individual skill improvement (56–58). Moreover, simulation training begins to move from virtual reality (VR) dependent to standardized patients, which have been already used in dermatology, emergency medicine, communication and counseling skills, mental health education, and social emotional development (59–65). In this phase, medical humanistic factors are essential to improve the outcomes of clinical patients, non-technical simulated training has made great progress when translated to clinical practice outcomes including palliative care (66). Meanwhile, objective structured clinical examination (OSCE) has been designed to assess the situation after the participants received simulated training some time ago (67–69). One of the distinctive features of research in this period is the attitude of the participants and the importance they attach to the simulation training. In another word, the feedback of the participants (70–74). The process from simulated training to clinical practice and then simulated training helps settle the problems in SBME and redesign the training program, and better realizes the clinical transformation and medical service.

### Research Trend and Future Direction Thinking

Understanding the latest research focus can help us quickly and effectively grasp the future direction of our research field to promote efficient development. From the burst words and related literature in recent years, it is obvious that SBME research gradually focuses on long-term impact as well as skill retention more than temporary skill improvement (75, 76). So it is time for SBME to improve according to participants' perception and attitude toward simulation training transformation and feedback. For researchers, in the next decade, it may be time to focus on the T4 stage of translational science, called collateral results, including cost effectiveness, skill retention, and systemic educational and patient care improvements. T4 can be achieved when researchers design studies and measure outcomes beyond educational and clinical variables and are prepared for unintended research outcomes (77–79). Further studies are required to assess the sustainability of skill improvement of the participants over time and more reliable evaluation systems should be designed based on existing research. More objective structured clinical examination should be designed to assess the diverse impact. With the outbreak of COVID-19 in recent years, offline intensive simulation training has been affected by the COVID-19 epidemic. An increasing number of simulated medical education centers are actively exploring the online computer program of SBME. Moreover, the translational outcomes of other important clinical skills still need to be further explored, and more randomized controlled trials are still required to provide more clinical evidence.

### Strengths and Limitations

First, scientometric methods were used to assess the history and the trend of the research on the clinical translational outcomes of SBME to identify the effectiveness of simulation-based training before the clinical performance. The three most common scientometric tools, including CiteSpace, VOSviewer, and a website platform, which have been widely used in bibliometric reviews (80), were used in our study. Hence, the data analysis was thought to be objective. Besides, our results consisted of the most comprehensive analysis, including nearly all aspects that appeared in this area, even co-authorship cooperation and stage evolution; thus, we were able to get an insight into this field and provide a valuable reference to researchers contributing to SBME. However, objectively, our study still had several limitations. For example, our data only came from the WoSCC without searching other databases such as Embase or PubMed, although the WoSCC is the most frequently used database in scientometric research. Furthermore, our data were analyzed using computer tools rather than manually selected, resulting in the existence of bias. As a remedy, some outcomes were selected again manually so that we could increase the accuracy of the conclusions and decrease the errors. These problems may be solved in the future with the development of software updates.

## Conclusion

This study has demonstrated the global trends in translational outcomes of SBME. We analyzed cooperative relations between countries and distributions of institutions and listed the most influential journals and authors in the field. By clustering the co-authorship visualization map, we clearly showed different research orientations of collaboration between authors. We further analyzed the evolution of research topics in this field via the top-cited reference, timeline view and keyword burstiness. In this decade, translational SBME research moved from the laboratory stage to the bedside clinical stage. The scope of application gradually becomes wider, from technical skills to non-technical skills. The simulation improved gradually, from VR simulation to standardized patients. The way of thinking about simulated medical education has also changed, from one-way to two-way feedback and improvement. Further research on the comprehensive impact, the collateral results of SBME, and a computer-based autonomous skill learning system development in the context of the epidemic will become a critical direction in the near future.

## Data Availability Statement

The original contributions presented in the study are included in the article/supplementary materials, further inquiries can be directed to the corresponding author.

## Author Contributions

YT and SY designed the study, recorded the bibliometric data with the application of different software, edited the data for the statistical work, and wrote the original draft. CY searched the publications as well as prepared the modified figures and tables. YT, SY, and CY edited and revised the manuscript. YX contributed to the methodology, supervision, and project administration of the study. All authors approved the final manuscript for submission and agreed to be accountable for the work.

## Funding

This work was supported by Novice Research Grant from the Society for Simulation in Health Care (Grant No. 54671).

## Conflict of Interest

The authors declare that the research was conducted in the absence of any commercial or financial relationships that could be construed as a potential conflict of interest.

## Publisher's Note

All claims expressed in this article are solely those of the authors and do not necessarily represent those of their affiliated organizations, or those of the publisher, the editors and the reviewers. Any product that may be evaluated in this article, or claim that may be made by its manufacturer, is not guaranteed or endorsed by the publisher.

## References

[1] De Dombal FT Horrocks JC Staniland JR Gill PW. Simulation of clinical diagnosis: a comparative study. Br Med J. (1971) 2:575–7. 10.1136/bmj.2.5761.575 5579197 PMC1795843

[2] Issenberg SB McGaghie WC Hart IR Mayer JW Felner JM Petrusa ER. Simulation technology for health care professional skills training and assessment. JAMA. (1999) 282:861–6. 10.1001/jama.282.9.861 10478693

[3] McGaghie WC Issenberg SB Petrusa ER Scalese RJ. A critical review of simulation-based medical education research: 2003-2009. Med Educ. (2010) 44:50–63. 10.1111/j.1365-2923.2009.03547.x 20078756

[4] McGaghie WC Issenberg SB Cohen ER Barsuk JH Wayne DB. Does simulation-based medical education with deliberate practice yield better results than traditional clinical education? A meta-analytic comparative review of the evidence. Acad Med. (2011) 86:706–11. 10.1097/ACM.0b013e318217e119 21512370 PMC3102783

[5] Price J Naik V Boodhwani M Brandys T Hendry P Lam BK. A randomized evaluation of simulation training on performance of vascular anastomosis on a high-fidelity *in vivo* model: the role of deliberate practice. J Thorac Cardiovasc Surg. (2011) 142:496–503. 10.1016/j.jtcvs.2011.05.015 21742349

[6] Kessler DO Auerbach M Pusic M Tunik MG Foltin JC. A randomized trial of simulation-based deliberate practice for infant lumbar puncture skills. Simul Healthc. (2011) 6:197–203. 10.1097/SIH.0b013e318216bfc1 21527870

[7] Sawyer T Sierocka-Castaneda A Chan D Berg B Lustik M Thompson M. Deliberate practice using simulation improves neonatal resuscitation performance. Simul Healthc. (2011) 6:327–36. 10.1097/SIH.0b013e31822b1307 21937960

[8] McGaghie WC. Medical education research as translational science. Sci Transl Med. (2010) 2:19cm8. 10.1126/scitranslmed.3000679 20371485

[9] Merigó JM Lafuente AMG Yager RR. An overview of fuzzy research with bibliometric indicators. Appl Soft Comput. (2015) 27:420–33. 10.1016/j.asoc.2014.10.035

[10] Chen C. Searching for intellectual turning points: Progressive knowledge domain visualization. Proc Natl Acad Sci USA. (2004) 101:5303–10. 10.1073/pnas.0307513100 14724295 PMC387312

[11] Bornmann L de Moya Anegón F Leydesdorff L. Do scientific advancements lean on the shoulders of giants? A bibliometric investigation of the ortega hypothesis. PLoS ONE. (2010) 5:e13327. 10.1371/journal.pone.0013327 20967252 PMC2954151

[12] Kokol P BlaŽun Vošner H Završnik J. Application of bibliometrics in medicine: a historical bibliometrics analysis. Health Info Libr J. (2021) 38:125–38. 10.1111/hir.12295 31995273

[13] Kokol P. Meta approaches in knowledge synthesis in nursing: a bibliometric analysis. Nurs Outlook. (2021) 69:815–25. 10.1016/j.outlook.2021.02.006 33814160

[14] Kokol P Zagoranski S Kokol M. Software development with scrum: a bibliometric analysis and profile. arXiv. (2021) abs/2103.01095.

[15] Kokol P Kokol M Zagoranski S. Machine learning on small size samples: a synthetic knowledge synthesis. arXiv. (2021) abs/2103.01002. 10.1177/00368504211029777 PMC10358596 35220816

[16] Chen CM. CiteSpace II: detecting and visualizing emerging trends and transient patterns in scientific literature. J Am Soc Inform Sci Technol. (2006) 57:359–77. 10.1002/asi.2031725855820

[17] Hirsch JE. An index to quantify an individual's scientific research output. Proc Natl Acad Sci USA. (2005) 102:16569–72. 10.1073/pnas.0507655102 16275915 PMC1283832

[18] Issenberg SB McGaghie WC Petrusa ER Lee Gordon D Scalese RJ. Features and uses of high-fidelity medical simulations that lead to effective learning: a BEME systematic review. Med Teach. (2005) 27:10–28. 10.1080/01421590500046924 16147767

[19] McGaghie WC Issenberg SB Petrusa ER Scalese RJ. Effect of practice on standardised learning outcomes in simulation-based medical education. Med Educ. (2006) 40:792–7. 10.1111/j.1365-2929.2006.02528.x 16869926

[20] Nishisaki A Nguyen J Colborn S Watson C Niles D Hales R. Evaluation of multidisciplinary simulation training on clinical performance and team behavior during tracheal intubation procedures in a pediatric intensive care unit. Pediatr Crit Care Med. (2011) 12:406–14. 10.1097/PCC.0b013e3181f52b2f 20935588

[21] Cheng A Brown LL Duff JP Davidson J Overly F Tofil NM. Improving cardiopulmonary resuscitation with a CPR feedback device and refresher simulations (CPR CARES study): a randomized clinical trial. JAMA Pediatr. (2015) 169:137–44. 10.1001/jamapediatrics.2014.2616 25531167

[22] Kurosawa H Ikeyama T Achuff P Perkel M Watson C Monachino A. A randomized, controlled trial of *in situ* pediatric advanced life support recertification (“pediatric advanced life support reconstructed”) compared with standard pediatric advanced life support recertification for ICU frontline providers^*^. Crit Care Med. (2014) 42:610–8. 10.1097/CCM.0000000000000024 24231759

[23] Todsen T Henriksen MV Kromann CB Konge L Eldrup J Ringsted C. Short- and long-term transfer of urethral catheterization skills from simulation training to performance on patients. BMC Med Educ. (2013) 13:29. 10.1186/1472-6920-13-29 23433258 PMC3598217

[24] Nilsson PM Russell L Ringsted C Hertz P Konge L. Simulation-based training in flexible fibreoptic intubation: a randomised study. Eur J Anaesthesiol. (2015) 32:609–14. 10.1097/EJA.0000000000000092 24809483

[25] West N Konge L Cayé-Thomasen P Sørensen MS Andersen SA. Peak and ceiling effects in final-product analysis of mastoidectomy performance. J Laryngol Otol. (2015) 129:1091–6. 10.1017/S0022215115002364 26391052

[26] Nilsson C Sorensen JL Konge L Westen M Stadeager M Ottesen B. Simulation-based camera navigation training in laparoscopy-a randomized trial. Surg Endosc. (2017) 31:2131–9. 10.1007/s00464-016-5210-5 27770252 PMC5411407

[27] Vilmann AS Norsk D Svendsen MBS Reinhold R Svendsen LB Park YS. Computerized feedback during colonoscopy training leads to improved performance: a randomized trial. Gastrointest Endosc. (2018) 88:869–76. 10.1016/j.gie.2018.07.008 30031803

[28] Østergaard ML Rue Nielsen K Albrecht-Beste E Kjær Ersbøll A Konge L Bachmann Nielsen M. Simulator training improves ultrasound scanning performance on patients: a randomized controlled trial. Eur Radiol. (2019) 29:3210–8. 10.1007/s00330-018-5923-z 30617476

[29] Bube S Dagnaes-Hansen J Mahmood O Rohrsted M Bjerrum F Salling L. Simulation-based training for flexible cystoscopy - a randomized trial comparing two approaches. Heliyon. (2020) 6:e03086. 10.1016/j.heliyon.2019.e03086 31922043 PMC6948262

[30] Zevin B Aggarwal R Grantcharov TP. Simulation-based training and learning curves in laparoscopic Roux-en-Y gastric bypass. Br J Surg. (2012) 99:887–95. 10.1002/bjs.8748 22511220

[31] Sevdalis N Hull L Birnbach DJ. Improving patient safety in the operating theatre and perioperative care: obstacles, interventions, and priorities for accelerating progress. Br J Anaesth. (2012) 109 (Suppl. 1):i3–16. 10.1093/bja/aes391 23242749

[32] Khan R Scaffidi MA Walsh CM Lin P Al-Mazroui A Chana B. Simulation-Based training of non-technical skills in colonoscopy: protocol for a randomized controlled trial. JMIR Res Protoc. (2017) 6:e153. 10.2196/resprot.7690 28778849 PMC5562936

[33] Grover SC Scaffidi MA Khan R Garg A Al-Mazroui A Alomani T. Progressive learning in endoscopy simulation training improves clinical performance: a blinded randomized trial. Gastrointest Endosc. (2017) 86:881–9. 10.1016/j.gie.2017.03.1529 28366440

[34] Walsh CM Scaffidi MA Khan R Arora A Gimpaya N Lin P. Non-technical skills curriculum incorporating simulation-based training improves performance in colonoscopy among novice endoscopists: randomized controlled trial. Dig Endosc. (2020) 32:940–8. 10.1111/den.13623 31912560

[35] Pucher PH Aggarwal R Singh P Tahir M Darzi A. Identifying quality markers and improvement measures for ward-based surgical care: a semistructured interview study. Am J Surg. (2015) 210:211–8. 10.1016/j.amjsurg.2014.11.013 25896316

[36] Pucher PH Aggarwal R Singh P Srisatkunam T Twaij A Darzi A. Ward simulation to improve surgical ward round performance: a randomized controlled trial of a simulation-based curriculum. Ann Surg. (2014) 260:236–43. 10.1097/SLA.0000000000000557 24646529

[37] Beaudoin PL Labbé M Fanous A Young M Rappaport J Park YS. Teaching communication skills to OTL-HNS residents: multisource feedback and simulated scenarios. J Otolaryngol Head Neck Surg. (2019) 48:8. 10.1186/s40463-019-0329-8 30691537 PMC6350291

[38] Varshney R Frenkiel S Nguyen LH Young M Del Maestro R Zeitouni A. The McGill simulator for endoscopic sinus surgery (MSESS): a validation study. J Otolaryngol Head Neck Surg. (2014) 43:40. 10.1186/s40463-014-0040-8 25927463 PMC4210497

[39] McGaghie WC Issenberg SB Barsuk JH Wayne DB. A critical review of simulation-based mastery learning with translational outcomes. Med Educ. (2014) 48:375–85. 10.1111/medu.12391 24606621

[40] McGaghie WC Draycott TJ Dunn WF Lopez CM Stefanidis D. Evaluating the impact of simulation on translational patient outcomes. Simul Healthc. (2011) 6(Suppl):S42–7. 10.1097/SIH.0b013e318222fde9 21705966 PMC3153601

[41] Andreatta P Saxton E Thompson M Annich G. Simulation-based mock codes significantly correlate with improved pediatric patient cardiopulmonary arrest survival rates. Pediatr Crit Care Med. (2011) 12:33–8. 10.1097/PCC.0b013e3181e89270 20581734

[42] Steinemann S Berg B Skinner A DiTulio A Anzelon K Terada K. *In situ*, multidisciplinary, simulation-based teamwork training improves early trauma care. J Surg Educ. (2011) 68:472–7. 10.1016/j.jsurg.2011.05.009 22000533

[43] Hunziker S Johansson AC Tschan F Semmer NK Rock L Howell MD. Teamwork and leadership in cardiopulmonary resuscitation. J Am Coll Cardiol. (2011) 57:2381–8. 10.1016/j.jacc.2011.03.017 21658557

[44] Graber ML Kissam S Payne VL Meyer AN Sorensen A Lenfestey N. Cognitive interventions to reduce diagnostic error: a narrative review. BMJ Qual Saf. (2012) 21:535–57. 10.1136/bmjqs-2011-000149 22543420

[45] Gordon M Darbyshire D Baker P. Non-technical skills training to enhance patient safety: a systematic review. Med Educ. (2012) 46:1042–54. 10.1111/j.1365-2923.2012.04343.x 23078681

[46] Curtis JR Back AL Ford DW Downey L Shannon SE Doorenbos AZ. Effect of communication skills training for residents and nurse practitioners on quality of communication with patients with serious illness: a randomized trial. JAMA. (2013) 310:2271–81. 10.1001/jama.2013.282081 24302090 PMC4310457

[47] Sarwani N Tappouni R Flemming D. Use of a simulation laboratory to train radiology residents in the management of acute radiologic emergencies. AJR Am J Roentgenol. (2012) 199:244–51. 10.2214/AJR.11.7892 22826384

[48] Sonnadara RR Garbedian S Safir O Mui C Mironova P Nousiainen M. Toronto orthopaedic boot camp III: examining the efficacy of student-regulated learning during an intensive, laboratory-based surgical skills course. Surgery. (2013) 154:29–33. 10.1016/j.surg.2013.05.003 23809482

[49] Barsuk JH McGaghie WC Cohen ER O'Leary KJ Wayne DB. Simulation-based mastery learning reduces complications during central venous catheter insertion in a medical intensive care unit. Crit Care Med. (2009) 37:2697–701. 10.1097/CCM.0b013e3181a57bc1 19885989

[50] Moura-Júnior LG Ramos A Campos JM Ferraz Á A Rocha HÂ L Costa GO. Teaching model for evaluation of the ability and competence progress in endosuture in surgical skill laboratory. Arq Bras Cir Dig. (2017) 30:256–9. 10.1590/0102-6720201700040007 29340549 PMC5793143

[51] Miranda A Kelly A Williams V Kelly M. Designing authentic simulations in ROSE and EBUS for undergraduate laboratory medicine students. BMJ Simul Technol Enhanc Learn. (2021) 7:97–101. 10.1136/bmjstel-2020-000597 35520377 PMC8936567

[52] Egle JP Malladi SV Gopinath N Mittal VK. Simulation training improves resident performance in hand-sewn vascular and bowel anastomoses. J Surg Educ. (2015) 72:291–6. 10.1016/j.jsurg.2014.09.005 25481803

[53] Cartier V Inan C Zingg W Delhumeau C Walder B Savoldelli GL. Simulation-based medical education training improves short and long-term competency in, and knowledge of central venous catheter insertion: a before and after intervention study. Eur J Anaesthesiol. (2016) 33:568–74. 10.1097/EJA.0000000000000423 27367432

[54] Nguyen N Elliott JO Watson WD Dominguez E. Simulation improves nontechnical skills performance of residents during the perioperative and intraoperative phases of surgery. J Surg Educ. (2015) 72:957–63. 10.1016/j.jsurg.2015.03.005 25911460

[55] Mahramus TL Penoyer DA Waterval EM Sole ML Bowe EM. Two hours of teamwork training improves teamwork in simulated cardiopulmonary arrest events. Clin Nurse Spec. (2016) 30:284–91. 10.1097/NUR.0000000000000237 27509565

[56] Fransen AF van de Ven J Banga FR Mol BWJ Oei SG. Multi-professional simulation-based team training in obstetric emergencies for improving patient outcomes and trainees' performance. Cochrane Database Syst Rev. (2020) 12:CD011545. 10.1002/14651858.CD011545.pub2 33325570 PMC8094450

[57] Barsuk JH Cohen ER Williams MV Scher J Jones SF Feinglass J. Simulation-Based mastery learning for thoracentesis skills improves patient outcomes: a randomized trial. Acad Med. (2018) 93:729–35. 10.1097/ACM.0000000000001965 29068818

[58] Gyan T Strobel NA McAuley K Shannon C Newton S Tawiah-Agyemang C. Health service provider education and/or training in infant male circumcision to improve short- and long-term morbidity outcomes: a systematic review. J Paediatr Child Health. (2019) 55:895–906. 10.1111/jpc.14528 31183922

[59] Spalding CN Rudinsky SL. Preparing emergency medicine residents to disclose medical error using standardized patients. West J Emerg Med. (2018) 19:211–5. 10.5811/westjem.2017.11.35309 29383083 PMC5785196

[60] Diaczok BJ Brennan S Levine D Hilu R Thati N Kruer J. Comparison of resident self-evaluation to standardized patient evaluators in a multi-institutional objective structured clinical examination: objectively measuring residents' communication and counseling skills. Simul Healthc. (2020) 15:69–74. 10.1097/SIH.0000000000000404 32044855

[61] Reger GM Norr AM Rizzo AS Sylvers P Peltan J Fischer D. Virtual standardized patients vs academic training for learning motivational interviewing skills in the US department of veterans affairs and the US military: a randomized trial. JAMA Netw Open. (2020) 3:e2017348. 10.1001/jamanetworkopen.2020.17348 33057643 PMC7563071

[62] Anderson OS Weirauch K Roper R Phillips J McCabe C Chuisano SA. The efficacy of hybrid telesimulation with standardized patients in teaching medical students clinical lactation skills: a pilot study. Breastfeed Med. (2021) 16:332–7. 10.1089/bfm.2020.0253 33493401

[63] Dietrich E Le Corre Y Dupin N Dréno B Cartier I Granry JC. Benefits of simulation using standardized patients for training dermatology residents in breaking bad news. Ann Dermatol Venereol. (2021) 148:156–60. 10.1016/j.annder.2020.11.003 33487487

[64] Reger GM Norr AM Gramlich MA Buchman JM. Virtual standardized patients for mental health education. Curr Psychiatry Rep. (2021) 23:57. 10.1007/s11920-021-01273-5 34268633

[65] Galal S Vyas D Mayberry J Rogan EL Patel S Ruda S. Use of standardized patient simulations to assess impact of motivational interviewing training on social? emotional development. Pharmacy. (2018) 6:65. 10.3390/pharmacy6030065 29997322 PMC6163181

[66] Kamdem VB Daelemans C Englert Y Morin F Sansregret A. Using simulation team training with human's factors components in obstetrics to improve patient outcome: a review of the literature. Euro J Obstetr Gynecol Reproduct Biol. (2021) 260:159–65. 10.1016/j.ejogrb.2021.03.015 33784580

[67] Nuzzo A Tran-Dinh A Courbebaisse M Peyre H Plaisance P Matet A. Improved clinical communication OSCE scores after simulation-based training: results of a comparative study. PLoS ONE. (2020) 15:e0238542. 10.1371/journal.pone.0238542 32886733 PMC7473530

[68] Hakimi M Kheirkhah M Abolghasemi J Hakimi R. Investigating the effect of neonatal resuscitation simulation using a competency-based approach on knowledge, skill, and self-confidence of midwifery students using objective structured clinical examination (OSCE). J Fam Med Prim Care. (2021) 10:1766–72. 10.4103/jfmpc.jfmpc_592_20 34123926 PMC8144786

[69] Albert DVF Ream M Verbeck N Lash T Weisleder P. An objective structured clinical examination of communication skills for child neurology residents. Pediatr Neurol. (2021) 114:68–74. 10.1016/j.pediatrneurol.2020.09.004 33242726

[70] Philippon AL Truchot J De Suremain N Renaud MC Petit A Baron GL. Medical students' perception of simulation-based assessment in emergency and paediatric medicine: a focus group study. BMC Med Educ. (2021) 21:586. 10.1186/s12909-021-02957-5 34798890 PMC8605506

[71] Jakobsen RB Gran SF Grimsmo B Arntzen K Fosse E Frich JC. Examining participant perceptions of an interprofessional simulation-based trauma team training for medical and nursing students. J Interprof Care. (2018) 32:80–8. 10.1080/13561820.2017.1376625 28985089

[72] Fawaz M Anshasi HA. Senior nursing student's perceptions of an interprofessional simulation-based education (IPSE): a qualitative study. Heliyon. (2019) 5:e02546. 10.1016/j.heliyon.2019.e02546 31667396 PMC6812179

[73] Dix S Morphet J Jones T Kiprillis N O'Halloran M Piper K. Perceptions of final year nursing students transer of clinical judgement skills from simulation to clinical practice: a qualitative study. Nurse Educ Pract. (2021) 56:103218. 10.1016/j.nepr.2021.103218 34619616

[74] Ahmed S Al-Mously N Al-Senani F Zafar M Ahmed M. Medical teachers' perception towards simulation-based medical education: a multicenter study in Saudi Arabia. Med Teach. (2016) 38 (Suppl. 1):S37–44. 10.3109/0142159X.2016.1142513 26984032

[75] Taylor S Avrith N Loo G Millán R Wyler BA McVane B. Impact of a focused trauma course on retention of provider skills, knowledge and confidence at a regional hospital in the Dominican Republic. Injury. (2021) 52:2526–33. 10.1016/j.injury.2021.06.001 34148653

[76] Menezes P Guraya SY Guraya SS. A systematic review of educational interventions and their impact on empathy and compassion of undergraduate medical students. Front Med. (2021) 8:758377. 10.3389/fmed.2021.758377 34820397 PMC8606887

[77] Tanya S Dubrowski A. Development of a cost-effective pediatric intubation task trainer for rural medical education. Cureus. (2020) 12:e6604. 10.7759/cureus.6604 32064186 PMC7008755

[78] Chuang M Purswani H Fazzari MJ Kaplan J Pardanani S Banks EH. A low-cost trainer for the surgical management of postpartum hemorrhage. Simul Healthc. (2020) 15:289–94. 10.1097/SIH.0000000000000434 32218092

[79] Rice MK Zenati MS Novak SM Al Abbas AI Zureikat AH Zeh HJ. Crowdsourced assessment of inanimate biotissue drills: a valid and cost-effective way to evaluate surgical trainees. J Surg Educ. (2019) 76:814–23. 10.1016/j.jsurg.2018.10.007 30472061

[80] Huang X Fan X Ying J Chen S. Emerging trends and research foci in gastrointestinal microbiome. J Transl Med. (2019) 17:67. 10.1186/s12967-019-1810-x 30819194 PMC6396506

